# Combined Olecranon Osteotomy and the Posterior Minimal Invasive Plate Osteosynthesis Approach for a Concomitant Injury of the Humeral Shaft and a Distal Intraarticular Humerus Fracture

**DOI:** 10.7759/cureus.5966

**Published:** 2019-10-22

**Authors:** Rahul Yadav, Mayur Nayak, Siddhartha Maredupaka, Mohammed Sadiq, Kamran Farooque

**Affiliations:** 1 Orthopaedics, All India Institute of Medical Sciences, New Delhi, IND; 2 Orthopaedics, Employees State Insurance Corporation Medical College, Gulbarga, IND

**Keywords:** complex distal humerus fracture, humerus shaft fracture, olecranon osteotomy, minimal invasive plate osteosynthesis

## Abstract

A complex fracture involving the distal humerus is a difficult fracture to treat and more so when it is involved with the ipsilateral shaft of the humerus. Open reduction and internal fixation of the humeral shaft with articular reconstruction have been described for a successful outcome of these complex fractures. However, it has drawbacks, especially in terms of soft tissue dissection and subsequent scarring and non-union.

A 42-year-old female presented to the emergency department with a fracture of the intercondylar humerus with an ipsilateral shaft of the left humerus. Combined olecranon osteotomy with posterior minimal plate osteosynthesis was used to treat this fracture. At the one-year follow-up at the postoperative fracture clinic, there was no pain, the range of motion (ROM) of the elbow was 10 degrees to 140 degrees and the radiograph showed a healed fracture with the implant in situ.

We present and review a novel technique to treat complex humerus fractures. Articular fragments can be directly visualized and fixed simultaneously. This approach allows for the biological fixation of the fracture and forms a reliable option for treating such complex fractures.

## Introduction

A fracture of the distal humerus involving the articular surface is one of the difficult fractures for an orthopedic surgeon. Complications such as bone loss, articular incongruity, non-union, malunion, and elbow stiffness are common [1]. When this injury is associated with a concomitant fracture of the ipsilateral shaft of the humerus, the complexity increases even further. Open reduction and internal fixation of the humeral shaft with articular reconstruction have been described in the past for a successful outcome of these complex fractures [2-3]. However, it has drawbacks, especially in terms of soft tissue dissection and subsequent scarring. The combined olecranon osteotomy with posterior minimal plate osteosynthesis (MIPO) described herein provides a reliable method for the treatment of these complex fractures.

## Case presentation

**Figure 1:**
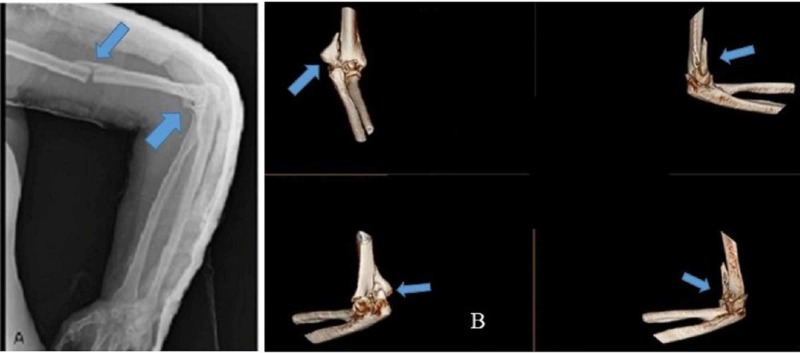
Preoperative radiograph showing the left shaft of humerus fracture with a concomitant distal humerus fracture (A). Computed tomography scan of the left distal humerus showing OTA/AO-13-C2 (Orthopaedic Trauma Association/Arbeitsgemeinschaft für Osteosynthesefragen) fracture with a Type III capitellar fracture (B).

Our patient, a 42-year-old, right hand dominated female was brought to the trauma center after a history of a fall from the first floor of a building. She presented with pain in the left arm and elbow. On examination, there was swelling and tenderness at the left mid-arm and left elbow and the neurovascular status was normal. Examination of the spine and lower limbs did not reveal any abnormality. A radiograph of the left upper extremity showed a transverse fracture of the mid-middle third-diaphyseal fracture of the humerus with a concomitant distal humerus fracture. A CT scan done for the elbow showed a comminuted fracture of the distal humerus (OTA/AO-13-C2) with a Type III capitellar fracture (Figure 1). The arm was immobilized in a splint, and the patient was shifted to the ward.

Surgical technique

**Figure 2:**
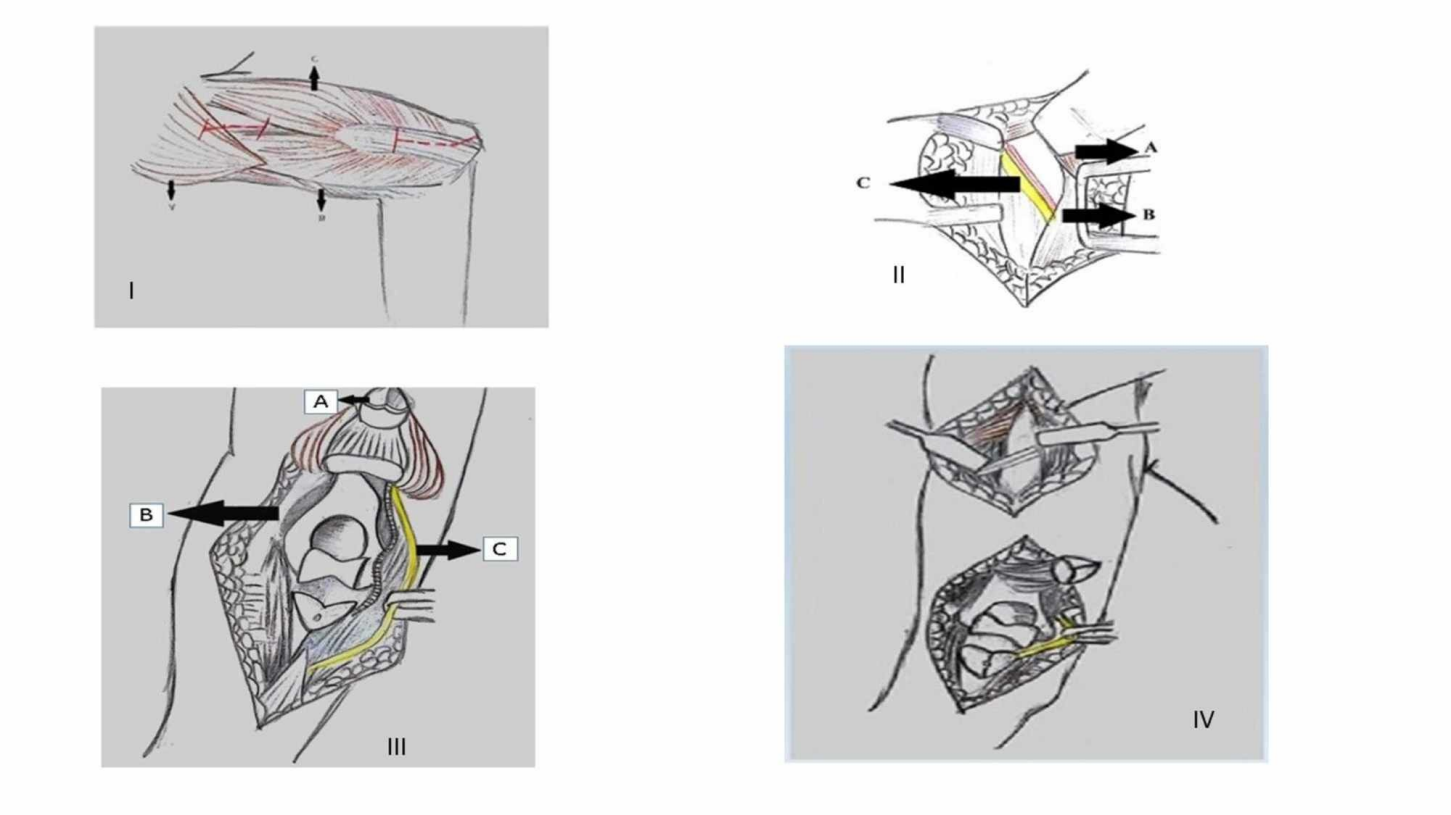
(I) Proximal incision between the middle and posterior deltoid raphe (A) and the long and lateral head of triceps surae (C). The distal incision starts 5 cm proximal to the tip of the olecranon was marked curving around the olecranon over the medial aspect, brachioradialis, and biceps brachii (B) (left arm depicted and viewed from superior). (II) Initial dissection carried in between the middle and posterior band of the deltoid muscle (A). Blunt dissection was carried out in between the two heads of triceps (B) and the radial nerve and the profunda brachii artery was located(C). (III) Distal dissection proceeds with an ulnar nerve transposition and chevron olecranon osteotomy, allowing complete articular surface exposure. Reflected olecranon osteotomy (A), ulnar nerve (B), and lateral intermuscular septum (C). (IV) Combined olecranon osteotomy with minimal invasive posterior osteosynthesis (MIPO). First distal humerus articular surface was reconstructed and then the plate was slid from the distal incision to the proximal incision.

**Figure 3:**
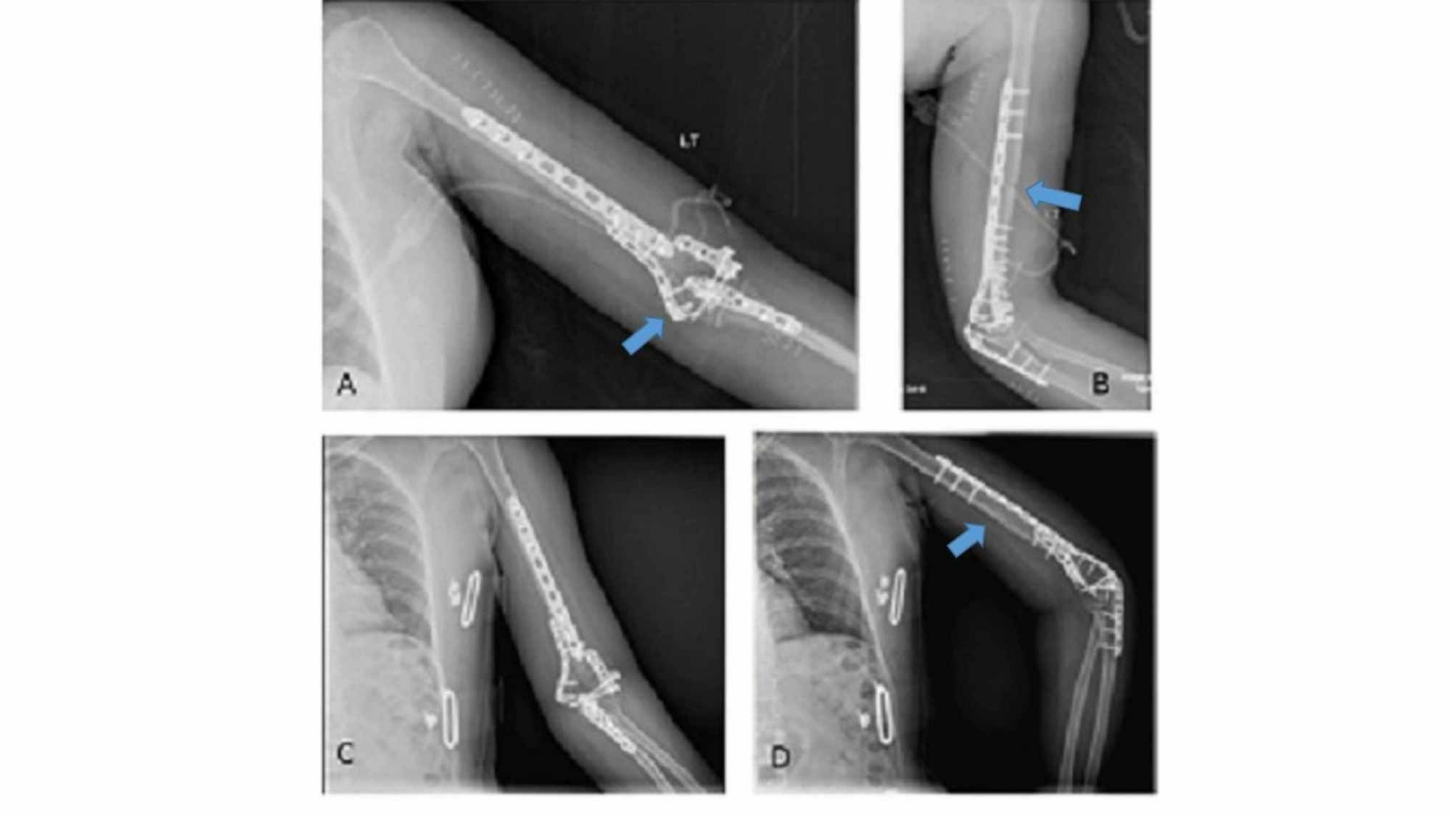
Anteroposterior (3A) and lateral (3B) radiograph showing fixation in the postoperative period and the healed fracture of shaft of the humerus (3C, 3D) at 12 months following surgery.

The rotation of the right arm was checked and recorded. The patient was positioned in a right lateral decubitus position following the administration of general anesthesia. The operative arm was placed at 90 degrees of forward elevation and internal rotation over a padded post such that the forearm could be flexed beyond 100 degrees. A 5 cm posterior incision was marked, starting 5 cm distal to the posterolateral acromion in line with the triceps. While approaching the olecranon, another incision starting 5 cm proximal to the tip of the olecranon was marked, curving around the olecranon over the medial aspect and ended up 5 cm distal to the tip of the olecranon over the subcutaneous border of the ulna. The incision was made along the marked line, and a full-thickness fasciocutaneous flap was developed (Figure 2). Dissection was carried out between the middle and posterior bands of the deltoid muscle along the posterior deltoid raphe. Blunt dissection was carried out in between the two heads, and the radial nerve was located. The nerve was released from the proximal to the distal direction so that the plate could be slid in an atraumatic fashion (Figure 2). On the distal aspect of the arm, the ulnar nerve was traced and isolated proximally until the medial intermuscular septum and distally until the first motor branch. Attention was then turned to the olecranon osteotomy. Submuscular exposure along the medial and lateral border of the ulna was performed, and apex distal chevron osteotomy was done according to the AO (Arbeitsgemeinschaft für Osteosynthesefragen) principles (Figure 2). Articular reconstruction for the distal humerus was performed, which was then rigidly fixed to the shaft in using a periarticular palate in the 90-90 fashion. The transverse mid-shaft fracture was then reduced in an indirect way, with the help of manual traction. A nine-hole 4.5 mm narrow combi-hole limited contact dynamic compression plate (LC-DCP) was slid from the distal to the proximal direction. The plate was placed along the posterior humeral surface deep to the radial nerve and the profunda brachii artery (Figure 2). The first screw placed was a 4.5 mm cortical screw in the distal hole of the plate. Thereafter, a 4.5 cortical screw was placed in the second-most proximal hole. The alignment was confirmed in the anteroposterior plane and the lateral plane under the c-arm. Clinically rotation of the injured limb was intraoperatively evaluated and confirmed. The rest of the screws were placed after verifying the plate position and securing proper alignment under the image intensifier. The olecranon osteotomy was fixed with an olecranon locking plate (Figure 3) and closure done in layers. Postoperatively, the patient did not have any neurological deficit and the elbow was mobilized with the help of continuous passive motion (CPM). The patient was routinely followed up in the clinic. At one year, the range of motion was 10 degrees to 140 degrees, and there was no restriction of activity (Figure 3).

## Discussion

Fracture of the humeral shaft associated with a distal humerus fracture is rare and difficult to approach. Typically, it requires an open reduction and internal fixation. Previously, Lewicky et al. [2] described a combined approach technique, the COLD (combined olecranon osteotomy, lateral paratricipital sparing, and deltoid insertion splitting) technique to deal with such kinds of fractures. In another report, Archdeacon MT et al. [3] described a triceps splitting approach with combined olecranon osteotomy for complex intercondylar fractures extending into the humeral diaphysis. Both of these techniques could have been used in our patient, however, owing to extensive dissection and periosteal stripping, we decided to use an olecranon osteotomy combined with posterior MIPO for the fracture.

In the present case, we developed an interval between the middle and posterior fibers of the deltoid with nerves preserved, which allowed us decreased traction on the axillary nerve, reduced the chance of injury to the superior lateral brachial cutaneous nerve, and prevented dissection of the anterior band of the deltoid. A proximal incision was planned according to the fact that the radial nerve traverses the humerus posteriorly at an average distance of 20.7 cm proximal to the medial epicondyle and 14.2 cm proximal to the lateral epicondyle.

Traditionally, a humerus fracture has been treated by open reduction and internal fixation via various surgical approaches. The complications include infection, non-union, iatrogenic radial nerve palsy, and refracture. One of the major problems with open plating is extensive soft tissue stripping and disruption of periosteal circulation, leading to these complications [2-3]. The MIPO technique offers a smaller incision and a procedure with lesser stripping, thus creating a conducive environment for better fracture healing. In the last few years, MIPO has gained popularity for the successful treatment of lower limb fractures [4-5] and has been described for humeral shaft fractures as well as via anterior and lateral approaches. We used a posterior MIPO approach, as described by Galluci et al. [6], because there was an associated distal humerus fracture for which we could extend the distal incision for the exposure of the distal humerus via olecranon osteotomy. However, we performed a deltoid split in our case in order to save the axillary nerve from traction injuries that might occur due to deltoid retraction. The intercondylar region is the most difficult area to approach while treating a distal humerus fracture for which we employed an olecranon osteotomy. Plate placement (orthogonal versus parallel) in a distal humerus fracture remains an issue of debate. Trikha et al. [7] recommended that the placement of the plate should be decided according to the fracture configuration. The orthogonal placement of the plate is especially beneficial in low supracondylar fractures. The present case had a comminuted distal humerus fracture (OTA 13-C3) along with a capitellar fracture, thus we went ahead with 90-90 plating with the help of pre-contoured LCP.

Although we performed a combined olecranon osteotomy with posterior MIPO, which has not been reported to the best of the author's knowledge, without any intraoperative or postoperative complication, it should be kept in mind that this approach may be associated with problems such as radial nerve palsy, malalignment of the fracture, and olecranon osteotomy non-union, which should be validated by further studies.

## Conclusions

In summary, our approach allows for the biological fixation of the humerus shaft fracture. Articular fragments can be directly visualized and fixed simultaneously. This technique involves less trauma and is a reliable option of treatment for fixing such complex fractures.
